# Dr. James Elliott Graham

**Published:** 1899-09

**Authors:** 


DR. JAMES ELLIOTT GRAHAM, M. D., M. R. C. P. Lond., of Toronto, died in that city July 7, 1899, aged 52 years. He was one of the most respected medical practitioners in Canada. He held many positions of professional importance, among which was that of professor of medicine and clinical medicine at the University of Toronto, which he filled at the time of his death. The *Canadian Practitioner* for August, 1899, publishes a well written memorial accompanied by an excellent portrait of this distinguished physician.

